# Prevalence and Factors Associated with Tongue Lesions Among Children Attending a University Dental Hospital in the Western Mediterranean Region of Türkiye 

**DOI:** 10.3290/j.ohpd.c_2775

**Published:** 2026-08-26

**Authors:** Derya Ceyhan, Gökçe Özcan Altınsoy

**Affiliations:** a Derya Ceyhan Associate Professor and Pediatric Dentist, Department of Pediatric Dentistry, Faculty of Dentistry, Suleyman Demirel University, Isparta, Türkiye. Conceived and designed the study, performed the clinical examinations, collected and analyzed the data, prepared the manuscript, contributed to the interpretation of the findings, critically revised the manuscript, and approved the final version.; b Gökçe Özcan Altınsoy Lecturer and Pediatric Dentist, Department of Pediatric Dentistry, Faculty of Dentistry, Antalya Bilim University, 07040 Antalya, Türkiye; Bilimdent Oral and Dental Health Center, Antalya Bilim University, 07190 Antalya, Türkiye. Performed the clinical examinations, collected and analyzed the data, prepared the manuscript, contributed to the interpretation of the findings, critically revised the manuscript, and approved the final version.

**Keywords:** children, fissured tongue, geographic tongue, prevalence, tongue lesions.

## Abstract

**Purpose:**

Tongue lesions in children are often overlooked during routine dental examinations despite their clinical relevance. This study evaluated the prevalence of tongue lesions among children attending a university dental hospital from the Western Mediterranean Region of Türkiye and explored their associations with demographic variables, systemic conditions, and oral hygiene–related factors.

**Materials and Methods:**

In this cross-sectional, clinic-based study, a total of 1097 children aged 2–13 years attending a pediatric dental clinic were included. Participants were categorized into two age groups (2–5 and 6–13 years). Tongue lesions were assessed through standardized oral examinations, and data on demographic characteristics, medical conditions, and oral hygiene practices were collected using a structured questionnaire. Associations between tongue lesions and these variables were analyzed using appropriate statistical tests.

**Results:**

Tongue lesions were detected in 6.2% of participants, with geographic tongue representing the most common lesion. Fissured tongue was more frequent in males overall (p=0.019) and in older children, while geographic tongue predominated in younger children. No statistically significant differences in tongue lesions were observed according to age groups or sex within age groups (p>0.05). Fissured tongue was associated with allergies in older children (p=0.039), whereas tongue lesions were more common in younger children with asthma (p=0.034). A positive family history of tongue lesions was statistically significantly more common among older and younger children with fissured tongue (p=0.001 and p=0.003, respectively), as well as among those with geographic tongue in both age groups (p=0.001 for both groups). Although tongue brushing was more common among older children (p=0.016), moderate and severe levels of tongue plaque were also higher in this group (p=0.040 and p=0.002, respectively). Tongue lesions (older children: DMFS, p=0.004 and DMFT, p=0.009; younger children: dfs, p=0.044 and dft, p=0.012), and higher plaque levels (older children: DMFS, p=0.020 and DMFT, p=0.022) were associated with increased caries indices, whereas mouthwash use was associated with lower caries indices (older children: DMFS, p=0.009 and DMFT, p=0.001).

**Conclusion:**

Tongue lesions may provide clinically relevant information regarding oral hygiene, caries experience, and systemic conditions, highlighting the importance of routine tongue examination in pediatric dental assessments.

Most oral lesions observed in pediatric populations are benign, whereas malignant lesions are exceedingly rare. These lesions may be asymptomatic or may present with pain, a burning sensation, irritation, obstruction, bleeding, or esthetic deformity.^15,31^ Lesions localized in the anterior region of the tongue do not interfere with breathing and swallowing functions, and symptoms are mild or absent. Diffuse lesions localized in the posterior region of the tongue may cause acute symptoms such as breathing or swallowing problems and may result in serious sequelae.^15^

Epidemiological studies have revealed that tongue lesions are frequently detected among oral mucosal lesions and that their prevalence varies across regions of the world.^2,18,30,32,35^ Considerable variation has been observed in the reported prevalence of tongue lesions among pediatric populations.^18,34,36^ Studies from different parts of the world have reported a wide range of prevalence rates of tongue lesions in children; for example, prevalence rates of approximately 24% to 39.7% have been reported in pediatric populations in Iran, while a rate of around 35% has been reported in Hungary.^20,29,36^ Similarly, studies conducted in Türkiye have reported prevalences of 4.9% and 11.7% in pediatric populations.^8,34^ Across these studies, fissured tongue and geographic tongue are consistently among the most commonly observed conditions, although other lesions such as ankyloglossia have also been reported.^8,20,29,34,36^ These differences may be attributed to variations in age, gender, ethnic origin, and socioeconomic status of the population studied, as well as to differences in diagnostic criteria, methodology, and sampling procedures used by different researchers.^7,32^ On the other hand, studies evaluating the frequency, types, and distribution of tongue lesions in pediatric populations remain limited, particularly in regional epidemiological settings.

The clinical appearance of tongue lesions varies, and the vast majority have a local etiology.^34^ However, tongue lesions are also considered reflections of the general health status of the human body. Recognizing tongue lesions may aid in the early diagnosis of hormonal, allergic, or systemic disorders and may be the first sign of certain diseases.^3,5,35^

Due to the influence of national and regional differences on oral lesions and their prevalence, and the limited number of studies conducted on this subject, this study aimed to determine the prevalence of tongue lesions in pediatric patients. Additionally, the associations between tongue lesions and age, gender, systemic conditions, sociodemographic and socioeconomic status, oral hygiene habits, and caries were evaluated.

## MATERIALS AND METHODS

For this study, ethical approval was obtained from the Institutional Clinical Research Ethics Committee of Faculty of Medicine at Suleyman Demirel University (Decision No: 2021/18-203).

Based on data from the literature, the prevalence of tongue lesions among 10,800 patients aged 2–13 years who attended our clinic within one year was estimated at 30%,^8,35,36^ and the minimum number of patients required in our study (α=0.05) was determined to be 311, using Neyman’s formula.^37^

**Fig 1:**
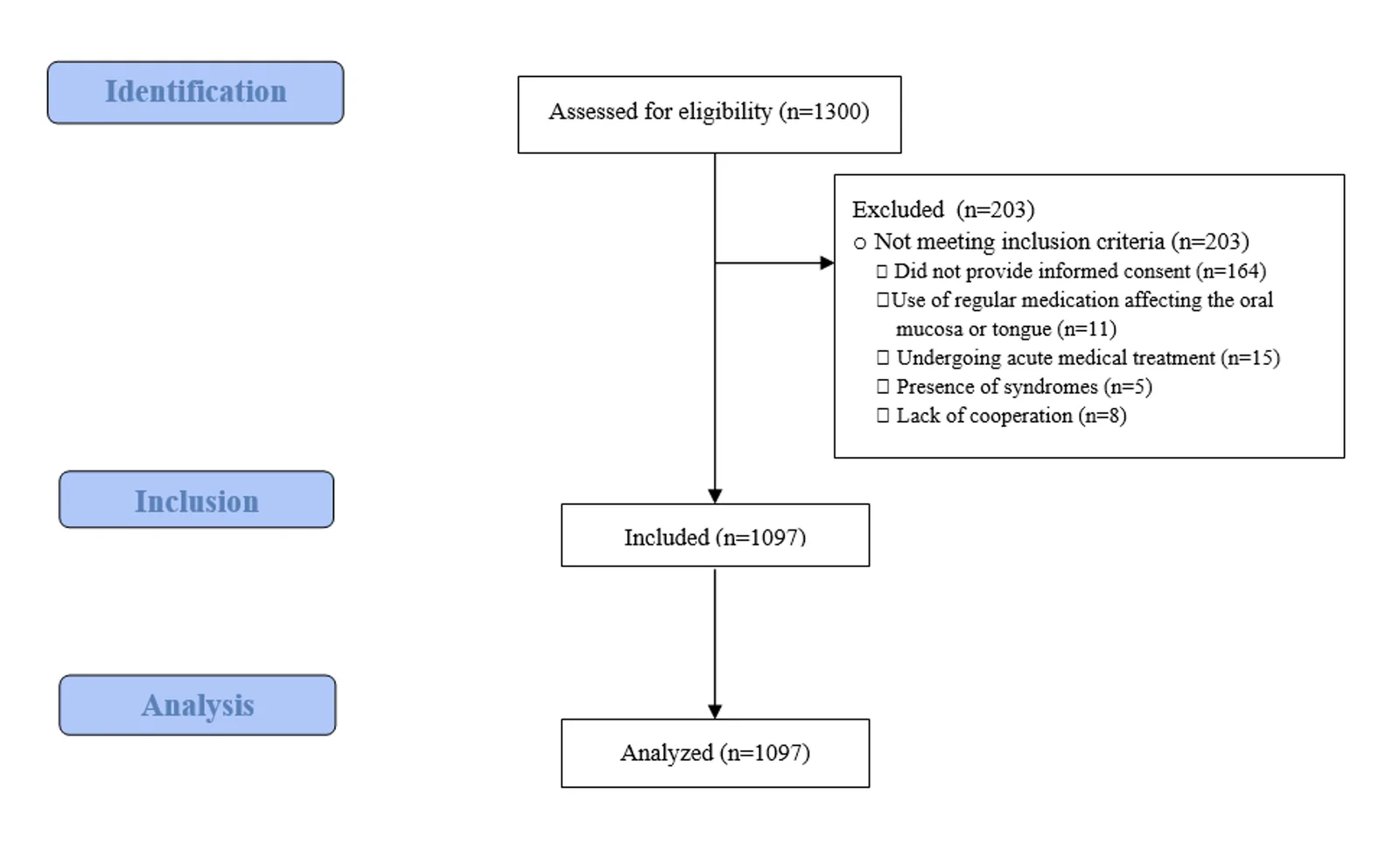
Flow diagram of participant selection of this cross-sectional study.

A total of 1097 pediatric patients aged 2–13 years, including 528 females and 569 males, who were brought to the pediatric dentistry clinic of a university hospital for routine examination and whose parents gave consent were consecutively recruited over a one-year period (Fig 1). Patients who did not provide consent for the study, who used regular medication that could affect the oral mucosa and tongue, who were receiving acute medical treatment, or who had any syndrome or cooperation problems were excluded from the study.

The sociodemographic status and medical histories of the patients included in the study were obtained. Routine clinical examinations of the patients were performed using a sterile mouth mirror, probe, and sponge under a reflector light at dental unit. Tongue examination was performed at rest position, with the mouth partially open, by pulling the tongue out with a sterile sponge. The tongue was evaluated for swelling, ulceration, lesions, surface and color changes, coating, size variations, mobility, and tongue margins.^21^ Patients were evaluated for geographic tongue, fissured tongue, median rhomboid glossitis, crenated tongue, traumatic fibroma, hairy tongue, coated tongue, ankyloglossia, macroglossia, and other lesions.^21,25^

Clinical examinations revealed decayed, and filled teeth (dft) and surfaces (dfs) in primary dentition, and decayed, missing, and filled teeth (DMFT) and surfaces (DMFS) in permanent dentition, occlusion status, and the presence or absence of any tongue lesions. Information was also gathered on the familial transmission of this lesion, its characteristics, symptoms, factors that trigger these symptoms, and measures to alleviate them, such as tongue brushing and mouthwash use. This information was obtained based on parental/self-reports and was not confirmed by clinical examination of family members.

The amount of plaque on the tongue was measured using Miyazaki’s tongue plaque index. According to this index, the plaque status on the tongue is scored based on the area covered by plaque: 0: no plaque; 1: less than one-third of the dorsal surface of the tongue is covered; 2: less than two-thirds of the dorsal surface of the tongue is covered; and 3: more than two-thirds of the dorsal surface of the tongue is covered.^28^

This information was recorded on forms specifically designed for age groups 2–5 and 6–13. The data were statistically analyzed by age group to assess the frequency of tongue lesions and the conditions associated with them.

The evaluations were performed by two researchers. Prior to the study, both researchers received standardized training to ensure consistency within themselves and between each other. One hundred patients with lesions to be evaluated in the study were included in the researchers’ calibration process, but were not included in the study. For these patients, who were evaluated independently at one-week intervals, intra- and inter-examiner reliability were assessed based on percentage agreement, yielding values of 100% and 98%, respectively. In cases of disagreement, the findings were discussed until a consensus was reached.

### Statistical Analysis

Data analysis was performed using SPSS 27.0 (SPSS, IBM; Armonk, NY, USA) and conducted at a 95% confidence level. The normality of the parameters was assessed using the Kolmogorov-Smirnov test, and it was determined that the parameters did not follow a normal distribution. While evaluating the study data, descriptive statistical methods (minimum, maximum, mean, standard deviation, median, frequency) were used, and for the comparison of quantitative data, Kruskal-Wallis test was used for the comparison of parameters belonging to more than two groups, and the Dunn test was used to identify the group causing the difference. The Mann-Whitney U test was used to compare parameters between two groups. The chi-squared test, Fisher’s Exact chi-squared test, and Fisher-Freeman-Halton Exact chi-squared test were used to compare qualitative data. Statistical significance was set at p<0.05.

## RESULTS

The 1097 patients in the study were evaluated in two age groups: 2–5 years (Group 2) and 6–13 years (Group 1). The mean age of all patients was 7.8±2.9 years, and the median age was 8 years. The mean age of children in the 2–5 age group was 4.13±0.95 years, with a median age of 4 years; the mean age of children in the 6–13 age group was 9.09±2.18 years, with a median age of 9 years. The sociodemographic data of the patients are presented in Table 1. Although the gender distribution ratios were similar, the proportion of males was higher in Group 1 and overall, while the proportion of females was higher in Group 2. The majority of participants in Group 1 and overall resided in Isparta, while those in Group 2 resided outside the city. The education level of both parents was mostly primary school. A total of 89.5% of patients had no medical problems. The most common medical problem in Group 1 was infectious disease, and in Group 2 was asthma.

**Table 1:** Sociodemographic data of the patients included in the study

Sociodemographic data		Group 1 6–13 years (n=811)		Group 2 2–5 years (n=286)		Total (n=1097)	
		n	%	n	%	n	%
Gender	Female	381	47.0	147	51.4	528	48.1
	Male	430	53.0	139	48.6	569	51.9
City of residence	Isparta	454	56.0	139	48.6	593	54.1
	Outside the city	357	44.0	147	51.4	504	45.9
Mother’s education level	Primary and middle school	398	49.1	131	45.8	529	48.2
	Highschool	269	33.2	95	33.2	364	33.2
	University	144	17.7	60	21.0	204	18.6
Father’s education level	Primary and middle school	310	38.2	108	37.8	418	38.1
	Highschool	280	34.5	108	37.8	388	35.4
	University	221	27.3	70	24.4	291	26.5
Medical problems	None	712	87.8	270	94.4	982	89.5
	Infectious diseases	33	4.1	5	1.8	38	3.5
	Asthma	14	1.7	7	2.4	21	1.9
	Allergy	24	3.0	2	0.7	26	2.4
	Systemic diseases	28	3.4	2	0.7	30	2.7


Tongue lesions were detected in 6.2% of the patients. The distribution of tongue lesions according to age groups is shown in Table 2. Geographic tongue was found in 26 of 1097 patients, and fissured tongue in 23. There was no statistically significant difference between age groups in terms of tongue lesions (p>0.05). In Group 1, fissured tongue and macroglossia were found together in 1 person, and geographic tongue and ankyloglossia were found together in 1 person. In Group 2, fissured and geographic tongue were found together in 1 person.

**Table 2:** The distribution of tongue lesions according to age groups

Tongue lesions	Group 1 (6–13 years) n (%)	Group 2 (2–5 years) n (%)	Total n (%)	p
Geographic tongue	18^b^ (2.2%)	8^c^ (2.8%)	26 (2.3%)	0.744
Fissured tongue	19^a^ (2.3%)	4^c^ (1.4%)	23 (2.0%)	0.473
Ankyloglossia	3^b^ (0.4%)	2 (0.7%)	5 (0.5%)	0.610
Crenated tongue	4 (0.5%)	0 (0%)	4 (0.4%)	0.578
Macroglossia	4^a^ (0.5%)	0 (0%)	4 (0.4%)	0.578
Coated tongue	4 (0.5%)	0 (0%)	4 (0.4%)	0.578
Median rhomboid glossitis	0 (0%)	1 (0.3%)	1 (0.1%)	0.261
Hairy tongue	1 (0.1%)	0 (0%)	1 (0.1%)	0.999
Traumatic fibroma	0 (0%)	0 (0%)	0 (0%)	x
Pigmented tongue	0 (0%)	0 (0%)	0 (0%)	x
Total	53 (6.5%)	15 (5.2%)	68 (6.2%)	0.515
*p<0.05 statistically significant; p>0.05 no significant relationship; chi-squared test; x: The test could not be performed. n = Number of cases with tongue lesions. ^a^ A patient with both fissured tongue and macroglossia. ^b^ A patient with geographic tongue and ankyloglossia. ^c^ A patient with both fissured and geographic tongue.				

When the data were examined regardless of group classification, the rate of fissured tongue in males (n=18, 3.2%) was statistically significantly higher than in females (n=5, 0.9%), (p=0.019). However, there was no statistically significant difference between males and females in the rate of other tongue lesions (p>0.05). When analyzed by age group, no statistically significant difference was found between males and females in the rate of any tongue lesion (p>0.05).

In Group 1, a statistically significant relationship was observed between medical conditions and the rate of fissured tongue, with a higher rate in children with allergies (n=2, 8.3%) compared to those without medical problems (n=14, 2.0%) (p=0.039). No statistically significant relationship was found between medical condition and other tongue lesions (p>0.05). In Group 2, a statistically significant relationship was found between medical condition and the presence or absence of tongue lesions. The rate of tongue lesions in children with asthma (n=2, 28.6%) was statistically significantly higher than in those without medical problems (n=11, 4.1%) (p=0.034). No statistically significant relationship was found between medical conditions and other tongue lesions (p>0.05).

No statistically significant differences were observed between age groups regarding the presence of symptoms related to tongue lesions or the factors triggering these symptoms (Table 3).

**Table 3:** Evaluation of the frequency of symptoms associated with tongue lesions according to age groups

Symptoms and trigger factors		Group 1 (6–13 years) (n=53)	Group 2 (2–5 years) (n=15)	p
		n (%)	n (%)	
Pain		2 (3.8%)	1^e^ (6.7%)	0.517
Burning sensation		1 (1.9%)	1^e^ (6.7%)	0.382
Bad breath		1 (1.9%)	0 (0%)	1.000
Other		2 (3.8%)	1 (6.7%)	0.517
Condition of having symptoms related to a tongue lesion (total)		6 (11.4%)	2 (13.4%)	0.674
Hot food		2^a^ (3.8%)	1^f^ (6.7%)	0.517
Salty food		4^a,b,c^ (7.5%)	2^f,g^ (13.3%)	0.600
Spicy food		4^a,b,c^ (7.5%)	2^f,g^ (13.3%)	0.600
After dental treatment		0 (0%)	1^f^ (6.7%)	0.212
Other		3^a,c^ (5.7%)	1^f^ (6.7%)	0.848
Factors that trigger the symptoms (total)		7 (13.2%)	2 (13.3%)	0.936
*p<0.05 statistically significant; p>0.05 no significant relationship; Fisher’s Exact test. n= Number of patients with symptoms/triggering factors associated with tongue lesions. ^a^ In 1 patient, hot, salty, spicy food, and other options were noted as triggering factors. ^b^ In 1 patient, salty and spicy foods were noted as triggering factors. ^c^ In 2 patients, salty, spicy foods, and other options were noted as triggering factors. ^e^ In 1 patient, pain and burning symptoms were observed together. ^f^ In 1 patient, each of the triggering factors has been reported. ^g^ In 1 patient, salty and spicy foods were noted as triggering factors.				

No statistically significant difference was found between children with and without fissured tongue in Group 1 in terms of symptoms related to tongue lesions (p>0.05). This situation was also observed in children with and without geographic tongue (p>0.05). In Group 1, 2 of the 19 patients with fissured tongue had symptoms related to tongue lesions; one had no specific symptoms, while the other had bad breath. In 4 of the 18 patients with geographic tongue, symptoms related to tongue lesions were observed; in 1, no specific symptom was detected; in 2, pain; and in the other, a burning sensation. In Group 2, symptoms related to tongue lesions were observed in 2 patients. In one of these two patients diagnosed with geographic tongue, pain and burning symptoms were observed together, while no specific symptom was found in the other. No significant difference was found between children with and without geographic tongue in terms of symptoms related to tongue lesions (p>0.05).

It was determined that there were 13 and 5 patients in Groups 1 and 2, respectively, who had tongue lesions in themselves and their families; 2 patients in both groups who did not have tongue lesions themselves but had them in their families; and 39 and 9 patients who had tongue lesions in themselves but not in their families. In Group 1, the rate of tongue lesions in the families of patients with fissured tongue (36.8%) was statistically significantly higher than that in the families of children without fissured tongue (1.0%); the rate of tongue lesions in the families of patients with geographic tongue (22.2%) was found to be statistically significantly higher than that in the families of patients without geographic tongue (1.4%) (p=0.001). The rate of tongue lesions in the families of patients with fissured tongue in Group 2 (50.0%) was statistically significantly higher than that in the families of patients without fissured tongue (1.8%); the rate of tongue lesions in the families of patients with geographic tongue (25.0%) was found to be statistically significantly higher than that in the families of patients without geographic tongue (1.8%) (p=0.003, p=0.001).

Tongue-brushing habits differed statistically significantly between the groups, with a higher rate observed in Group 1 (p=0.016). Moderate and severe tongue plaque levels were also statistically significantly higher in Group 1 (p=0.040, p=0.002) (Table 4). In Group 2, patients with fissured tongue showed lower rates of mild plaque but higher rates of severe plaque compared to those without the lesion (p=0.042).

**Table 4:** Findings related to oral hygiene by age group

Oral hygiene habits and status		Group 1 6–13 years (n=811)		Group 2 2–5 years (n=286)		Total (n=1097)		p
		n	%	n	%	n	%	
Mouthwash use	No	776	95.7	0	0	776	95.7	x
	Yes	35	4.3	0	0	35	4.3	
Mouthwash use frequency	No	776	95.7	0	0	776	95.7	x
	More than once a day	13	1.6	0	0	13	1.6	
	Once a day	9	1.1	0	0	9	1.1	
	Once a week	3	0.4	0	0	3	0.4	
Tongue-brushing status	No	751	92.6	277	96.9	1028	93.7	0.016*
	Yes	60	7.4	9	3.1	69	6.3	
Mild plaque on the tongue	No	376	46.4	130	45.5	506	46.1	0.791
	Yes	435	53.6	156	54.5	591	53.9	
Moderate plaque on the tongue	No	625	77.1	237	82.9	862	78.6	0.040*
	Yes	186	22.9	49	17.1	235	21.4	
Severe plaque on the tongue	No	768	94.7	283	99.0	1051	95.8	0.002*
	Yes	43	5.3	3	1.0	46	4.2	
*p<0.05 statistically significant; p>0.05 no statistically significant relationship; chi-squared test. x: The test could not be performed.								

When evaluating sagittal occlusal relationships of the upper and lower teeth, Class I occlusion was observed in 84.6% of patients in Group 1, Class II in 10.1%, and Class III in 5.3%. When vertical and transverse occlusal relationships were evaluated, 2.8% of patients had an edge-to-edge bite, 0.6% had an open bite, 1.5% had a deep bite, and 2.7% had a crossbite. In Group 2 patients, the sagittal occlusal relationships between the second primary molars were: a mesial step in 51.7%, a distal step in 4.5%, and a terminal plane relationship in 43.7%. An open bite was detected in 1% of these patients, a deep bite in 0.7%, and a crossbite in 0.3%. In Group 2, mild plaque presence on the tongue was more common in patients with mesial step and terminal plane; moderate plaque presence was more common in patients with distal step (p=0.008; p=0.001). No statistically significant relationship was found between occlusal relationships and the presence of fissured tongue, geographic tongue, and tongue lesions in Groups 1 and 2 (p>0.05).

The mean, minimum, maximum, and median values for the DMFT/dft and DMFS/dfs indices of patients in Group 1 were found to be 1.01±1.69 (0-12, 0), 2.95±3.21 (0-16, 2), 1.36±2.55 (0-19, 0), and 5.64±8.17 (0-55, 3), respectively. The mean, minimum, maximum, and median values for the dft and dfs indices in Group 2 were 6.5±4.92 (0-20, 6) and 10.77±10.04 (0-74, 8), respectively. The mean, minimum, maximum, and median values for the total DMFT/dft and DMFS/dfs indices were 1.01±1.69 (0-12, 0), 3.76±3.70 (0-20, 3), 1.36±2.55 (0-19, 0), and 6.98±8.98 (0-74, 4), respectively.

In Group 1, DMFS and DMFT values were statistically significantly higher in patients with tongue lesions and in those with symptoms related to these lesions (p<0.05). Mouthwash use was associated with statistically significantly lower DMFS and DMFT values (p<0.05). In addition, higher tongue plaque levels were associated with increased values of DMFS and DMFT indices (p<0.05). In Group 2, patients with tongue lesions had statistically significantly higher dfs and dft values compared to those without lesions (p<0.05) (Table 5).

**Table 5:** The comparison of caries index values across groups based on tongue lesions, symptoms, plaque level, and mouthwash use

Group 1 (6–13 years)		DMFS	dfs	DMFT	dft
		Mean±SS (median)	Mean±SS (median)	Mean±SS (median)	Mean±SS (median)
Group 2 (2–5 years)					
Tongue lesion	No	1.3±2.54 (0)	5.55±8.1 (3)	0.98±1.68 (0)	2.92±3.17 (2)
	Yes	2.1±2.64 (1)	6.96±9.13 (4)	1.46±1.74 (1)	3.38±3.67 (2)
	1p	0.004*	0.372	0.009*	0.502
Symptoms related to the tongue lesion	No	1.78±2.44 (0.5)	7.02±9.59 (3)	1.24±1.57 (0.5)	3.39±3.84 (2)
	Yes	4.5±3.08 (5)	6.5±4.76 (7.5)	3.17±2.23 (3)	3.33±2.16 (3.5)
	1p	0.041*	0.547	0.035*	0.665
Mouthwash use	No	1.4±2.59 (0)	5.66±8.17 (3)	1.05±1.71 (0)	2.97±3.21 (2)
	Yes	0.37±0.94 (0)	5.26±8.25 (1)	0.17±0.51 (0)	2.57±3.26 (1)
	1p	0.009*	0.381	0.001*	0.324
Level of plaque on the tongue	None	1.46±2.66 (0)ab	6.28±9.45 (3)a	1.02±1.63 (0)ab	3.13±3.35 (2)a
	Mild	1.1±2.2 (0)a	5.89±8.44 (3)a	0.87±1.54 (0)a	3.03±3.25 (2)a
	Moderate	1.82±3.2 (0)b	4.77±6.71 (2)a	1.28±2.03 (0)b	2.64±3.09 (1)a
	Severe	1.56±2.03 (1)b	4.7±5.97 (3)a	1.3±1.57 (1)b	2.86±2.73 (3)a
	2p	0.020*	0.655	0.022*	0.549
Tongue lesion	No		10.57±10.02 (8)		5.92±4.01 (6)
	Yes		14.64±10.1 (10.5)		8.57±3.5 (8)
	1p		0.044*		0.012*
*p<0.05 statistically significant; p>0.05 no statistically significant relationship; 1Mann-Whitney U test; 2Kruskal-Wallis test. Different lower-case letters in the columns indicate the differences between the groups in the relevant groups.					

## DISCUSSION

There are few reports in the literature on oral mucosal disorders in children and tongue lesions as a component of these disorders.^8,34,35,36^ The lack of epidemiological data on this subject may lead to the neglect of the tongue, an important organ of the oral cavity, and its diseases and possible associations. Therefore, our study aimed to determine the prevalence of tongue lesions in pediatric patients and the associations between these lesions and age, gender, systemic conditions, sociodemographic and socioeconomic status, oral hygiene habits, and caries.

In our study, tongue lesions were detected in 6.2% of 1097 children aged 2–13 years. In a study examining 1017 children aged 1–14 years in Hungary, this rate was 35.1%.^36^ In a study conducted on 800 children aged 3–14 years in Türkiye, the rate was 11.7%.^8^ In Iran, the rate was 24.3% in 1540 children aged 7–17 years and 39.7% in 1600 children aged 6–12 years.^20,29^ It should be noted that these rates, which vary across different regions of the world, may be due to differences in the sample groups’ race, gender, and age; the types of tongue lesions evaluated; the cultural habits of the population; and differences in the diagnostic criteria, methods, and procedures used by researchers.

Our study found that the most common tongue lesion across all age groups was geographic tongue (2.3%), followed by fissured tongue (2.0%). Studies conducted in Hungary and Türkiye on children aged 0–14 years showed that the most common lesion was fissured tongue (29.2%, 3.4%, and 7.5%, respectively).^8,35,36^ Considering age groups in our study, fissured tongue was the most common finding in Group 1 (6–13 years old) (2.3%), followed by geographic tongue (2.2%). In Group 2 (2–5 years old), geographic tongue was the most common (2.8%), followed by fissured tongue (1.4%). When studies with a similar age range to Group 1 were examined, the prevalence of geographic tongue ranged from 1.8% to 27%.^20,29,34^ In studies conducted with children of a similar age range to Group 2, the prevalence rates of geographic tongue were 1.6%, 21%, and 36.5%, respectively.^1,3,4^ Studies from Türkiye, including age ranges comparable to Group 1, reported fissured tongue prevalence rates between 7.3% and 14%, whereas studies involving age groups similar to Group 2 reported lower rates of 1.1% and 1.7%.^8,35^

In a study examining adults in the United States, geographic tongue was found to be associated with ethnic origin.^33^ It has been suggested that geographic tongue can present in different forms, and if only a single examination is performed to investigate this lesion, especially in atypical cases, a large number of patients may be assessed as unaffected.^16^ At the same time, longitudinal rather than cross-sectional studies are needed to determine the true prevalence rate, given its variable nature. It has been suggested that this situation may also affect the study’s results.^7^ Furthermore, many studies have indicated that geographic tongue decreases with age.^7,8^ Although the etiology of geographic tongue remains unknown, various hypotheses have been proposed. Its prevalence is higher among the parents and siblings of individuals with this anomaly, and the presence of the lesion in at least one parent further increases it among siblings. Therefore, it has been suggested that it may be transmitted through polygenic inheritance, possibly via familial transmission.^10^ Major histocompatibility complexes have been shown to play a role in the pathogenic mechanism of geographic tongue and to share common genetic determinants with psoriasis.^14^ The literature indicates that celiac disease, Reiter’s disease, allergies, gastrointestinal disorders, oral contraceptive use, oral microbiome, asthma, stress, and juvenile diabetes may be associated with geographic tongue.^6,13,27,38^

The etiology of fissured tongue is not yet clearly understood. It has been suggested that fissured tongue is frequently observed in patients with Down syndrome, psoriasis, vitamin B12 deficiency, and asthma.^13,23^ Histopathologically, inflammation is considered to be an important part of the disease, capable of altering symptoms and findings, and it is thought that an inflammatory response involving lymphocytes in the connective tissue plays a role in patients with fissured tongue.^22^ Low IgG levels in individuals with fissured tongue have been suggested to indicate a disorder in immunoglobulin synthesis/metabolism.^23^ The relationship between a fissured tongue and human leukocyte antigens (HLA) has been investigated, and several HLA molecules have been identified as associated with it.^19^ Fissured tongue is thought to be inherited in a polygenic manner.^10^ Although the pathogenic factors determining the age of onset and rate of progression are unknown, it has been observed that fissured tongue appears particularly after the age of 4 and before adolescence, and that the number of people affected by this condition and its severity increase with age.^24^ Studies have suggested that the prevalence of fissured tongue increases with age and, consequently, that fissured tongue is not congenital.^2,7,8,36^ It has been suggested that many oral lesions tend to develop more rapidly and frequently in elderly and adult individuals, and that the reasons for this tendency may include a decrease in immune response, a decrease in DNA repair capacity, and age-related atrophy of oral tissues, including the epithelium and salivary glands, as well as mineral and vitamin deficiencies.^9,12^

The relationship between tongue lesions and gender has also been frequently investigated. In our study, evaluated by age group, no statistically significant difference in the rate of tongue lesions was observed between males and females. This finding is consistent with studies conducted in Iran, Türkiye, and South Africa, which found no statistically significant association between tongue lesion prevalence and gender.^1,20,35^ In the present study, when evaluated without age-group differentiation, there was no statistically significant difference in the frequency of geographic tongue between genders. In this regard, it is similar to the results of a study conducted in Poland that examined a similar age group.^30^ Unlike the present findings, a Hungarian study reported an association between the prevalence of geographic tongue and gender, with males more commonly affected.^36^ In a study conducted in the Eastern Anatolia Region of Türkiye, geographic tongue was more common in females.^8^ This situation may be related to the population’s genetic characteristics and ethnicity. In addition, since geographic tongue can present in different forms, diagnosing based on a single examination may have yielded different results across studies. In our study, when evaluated without age-group differentiation, the rate of fissured tongue was statistically significantly higher in males than that in females. Studies conducted in Hungary, Türkiye, Nigeria, Jordan, and Switzerland show similar findings in this regard.^7,8,12,36^ Studies conducted in Iran and Afghanistan, however, showed that it was more common among females.^9,29^ These differences in the results may be attributable to genetic characteristics and ethnicity of the study population, as evidenced by the case of geographic tongue. The influence of disparate social living conditions is also a potential contributing factor.

It is known that tongue lesions may be associated with various medical conditions. In a study conducted in southern Iran involving 100 asthmatic patients aged 12–83 years, the most common oral lesions were fissured tongue (13%) and geographic tongue (10%).^13^ Studies involving children aged 0–13 years in Türkiye and 0–12 years in Brazil have suggested an association between allergy and fissured tongue,^3,35^ while studies examining individuals aged 1–14 years in Hungary and 4–60 years in Türkiye have identified an association between allergy and geographic tongue.^27,36^ In this current study, similarly, the rate of any tongue lesion in children aged 2–5 years with asthma and the rate of fissured tongue in children aged 6–13 years with allergies were statistically significantly higher than those in children without medical problems. The increased prevalence of these conditions in immune-mediated diseases such as asthma and allergies may be related to the inflammatory nature of tongue lesions, including geographic and fissured tongue. Atopy is a hereditary predisposition of the immune system to IgE-mediated hypersensitivity reactions in response to common environmental antigens. One study has shown that patients with geographic tongue exhibit increased levels of salivary IgE and eosinophilic cationic protein, an allergic inflammatory mediator, thereby suggesting a relationship between geographic tongue and allergic reactions, although this has not been conclusively demonstrated.^11^

In our study, geographic tongue and ankyloglossia were observed in one person in Group 1, while fissured and geographic tongue were observed together in one person in Group 2. In two studies conducted in Türkiye, the rate of geographic tongue accompanying fissured tongue was 0.8% and 0.6%,^8,35^ and geographic tongue and ankyloglossia have been observed concurrently in one patient, as in our study.^8^ In a study conducted on children in Hamadan, Iran, geographic tongue and fissured tongue coexisted in 77.2% of cases.^29^ In a Hungarian study, geographic tongue was found in 8.75% of children with fissured tongue, while fissured tongue was found in 44.82% of children with geographic tongue.^36^ Fissured and geographic tongue are considered polygenic conditions, and a statistically significant association between the two has been demonstrated.^10^ These two tongue lesions have been proposed as genetically related conditions that may represent different manifestations of the same disease, with geographic tongue potentially reflecting a stage in the progression of fissured tongue, thereby supporting the rationale for their joint evaluation.^24^ The finding in our study that the rate of tongue lesions in the families of children with fissured and/or geographic tongue is statistically significantly higher than that in the families of children without fissured and/or geographic tongue corroborates these findings.

In our study, no statistically significant differences in tongue-lesion-related symptoms were found between children with and without geographic tongue, or between children with and without fissured tongue. Geographic tongue is generally considered asymptomatic; however, some patients may experience a burning sensation. This has been attributed to increased sensitivity in areas of the dorsal tongue where papillae are lost, particularly to acidic foods.^7,17^ Fissured tongue is also generally considered asymptomatic, but if the fissures are deep enough to trap food debris, these areas can become a reservoir for bacteria and subsequently become inflamed, leading to symptoms.^7^

When evaluated for oral hygiene habits, children in Group 1 showed higher tongue-brushing frequency than those in Group 2. However, moderate and severe plaque levels on the tongue were more prevalent in Group 1. It is expected that children in Group 1, who are in an older age group, would have tongue brushing habits, and our results support this. On the other hand, the higher rate of plaque level may be due to ineffective tongue brushing. In children with fissured tongue in Group 2, a higher rate of severe tongue plaque was observed. One study identified a statistically significant association between the absence of tongue-brushing habits and fissured tongue, while also emphasizing that tongue-brushing practices are not a causal factor in the development of fissured tongue and that further research is required.^26^ In a study examining 906 children aged 6–12 years in Türkiye, no correlation was found between tongue lesions and oral hygiene.^34^ The higher values of caries indices observed in children with tongue lesions in our study groups may indicate that, depending on the nature of the lesion, more accumulation space may be created for microorganisms in the mouth, thereby establishing a relationship with plaque accumulation.

Despite its contributions, this study has certain limitations. First, the study population was derived from a single-center, clinic-based sample of patients presenting for care, which may limit the generalizability of the findings to the broader pediatric population and introduce potential bias in prevalence estimates. In addition, the cross-sectional design based on a single clinical examination limits causal inference between tongue lesions and associated factors. Finally, some variables—particularly information regarding family history of tongue lesions, associated symptoms, triggering factors, and related oral hygiene practices (e.g., tongue brushing and mouthwash use)—were obtained based on parental/self-reports and were not clinically verified, which may have introduced reporting bias.

Dentists are responsible for the health of not only the teeth in the oral cavity but also the tongue, an important component of oral health. The findings of the present study highlight the clinical importance of routine tongue examination in pediatric dental practice. The observed associations between tongue lesions, oral hygiene status, and caries indices suggest that these conditions may be interrelated in pediatric patients. From a preventive dentistry perspective, incorporating tongue assessment into routine clinical examinations may support early detection of oral mucosal alterations, facilitate targeted oral hygiene interventions, and contribute to improved monitoring of pediatric patients. In addition, increased awareness of tongue lesions may aid in identifying underlying lifestyle, dietary, or systemic factors and support appropriate referral when necessary.

## CONCLUSION

Tongue lesions may provide clinically relevant information regarding oral hygiene, caries experience, and systemic conditions in pediatric patients. These findings highlight the importance of incorporating routine tongue examination into comprehensive pediatric dental assessments, as it may facilitate the early recognition of oral mucosal alterations and support preventive care and appropriate clinical management.

### Data Availability Statement

The data supporting the findings of this study are not publicly available due to ethical and privacy restrictions on pediatric patient data. De-identified data may be made available from the corresponding author upon reasonable request and with permission from the relevant institutional authority.

## REFERENCES 
